# Crystal structure of hexa­chloro­thallate within a caesium chloride–phospho­tungstate lattice Cs_9_(TlCl_6_)(PW_12_O_40_)_2_·9CsCl

**DOI:** 10.1107/S2056989024005565

**Published:** 2024-06-14

**Authors:** Gauthier Deblonde, Ian Colliard

**Affiliations:** a Physical and Life Sciences Directorate Glenn T Seaborg Institute Lawrence Livermore National Laboratory,Livermore California 94550 USA; University of Hyogo, Japan

**Keywords:** crystal structure, polyoxometalates, thallium, extended framework

## Abstract

Crystal formation of caesium thallium chloride phospho­tungstates, Cs_9_(TlCl_6_)(PW_12_O_40_)_2_·9CsCl showcases the ability to capture and crystallize octa­hedral complexes *via* the use of polyoxometalates (POMs). The large number of caesium chlorides allows for the POM [α-PW_12_O_40_]^3−^ to arrange itself in a cubic close-packing lattice extended framework, in which the voids created enable the capture of the [TlCl_6_]^3−^ complex.

## Abstract 2

The cystallization and capture of hexa­chloro­thallium within the phospho­tunsgate and caesium chloride matrix is described.

## 1. Chemical context

The Keggin ion, [α-*X*W_12_O_40_]^*n*−^ (*X* = B, Si, P, Ga, Ge, *etc*.), along with many other polyoxometalates (POMs), are renowned for their ability to lose [WO_*x*_] moieties, yielding lacunary POMs (Pope, 1983 ▸). These lacunary derivatives of the Keggin, [*X*W_11_O_39_]^*n*−^, [*X*W_10_O_36_]^*n*−^, and [*X*W_9_O_34_]^*n*−^, have been extensively studied as chelators for metal ions, in which they directly bind to cations, for example trivalent lanthanides and actinides (Wang *et al.*, 2024 ▸). Recently, crystallization of microgram qu­anti­ties of the radioactive element curium (Cm^3+^) with [*X*W_11_O_39_]^*n*−^, showcased the utility of the POM chelators (Colliard *et al.*, 2022 ▸). However, not all metal ions have been able to coordinate to lacunary Keggin deriv­atives. In particular, some metals form highly stable complexes with smaller ions, like chlorides, impeding their potential inter­actions with POM chelators. As such, a new method to capture metal ions has been developed where the metal of choice can be captured in the lattice arrangement of the parent Keggin ion, [α-*X*W_12_O_40_]^*n*−^, instead of direct inter­action with lacunary Keggin ion ([*X*W_11_O_39_]^*n*−^). This allows for the POM-induced crystallization of the halide metal complex.

## 2. Structural commentary

**Figure 1:**
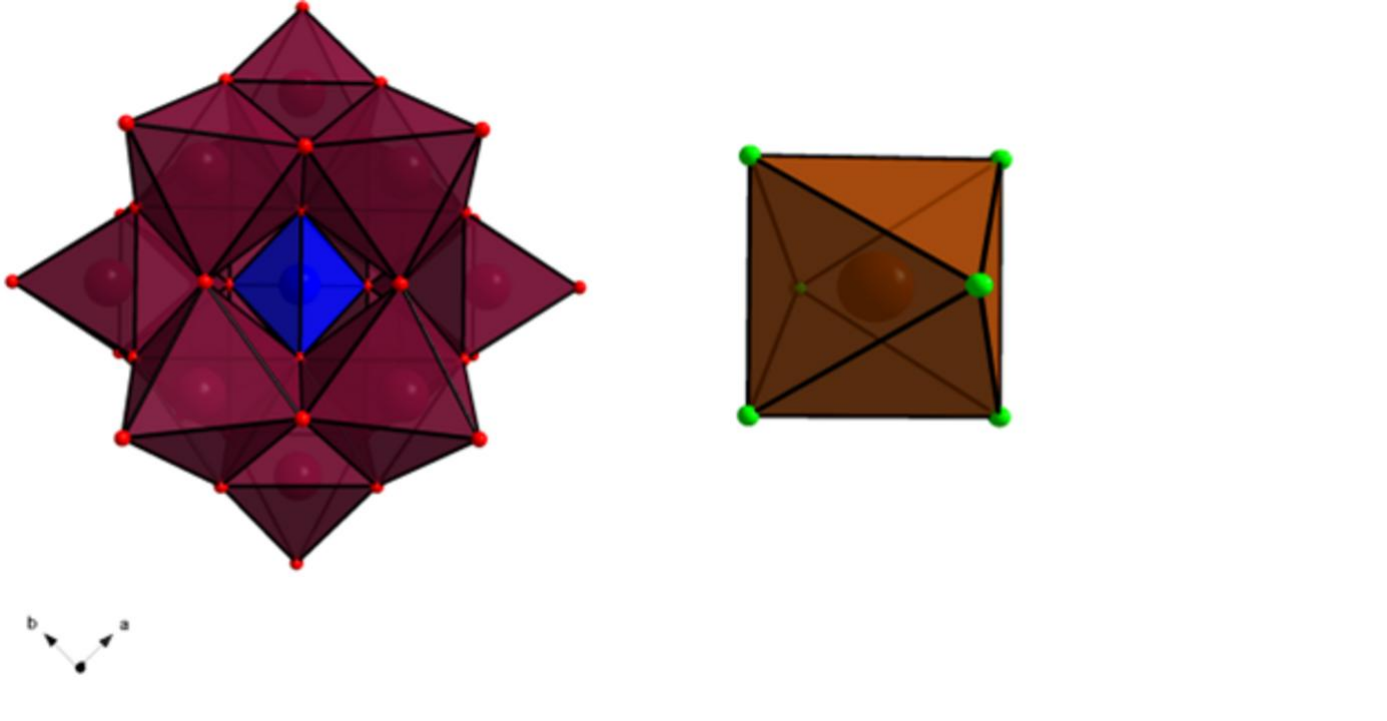
[Polyhedral representation of the Keggin ion, [α-PW_12_O_40_]^3−^ (left), and [TlCl_6_]^3−^ (right). W in maroon, O in red, P in blue, Cl in green and Tl in brown, with excess CsCl omitted for clarity]

**Figure 2:**
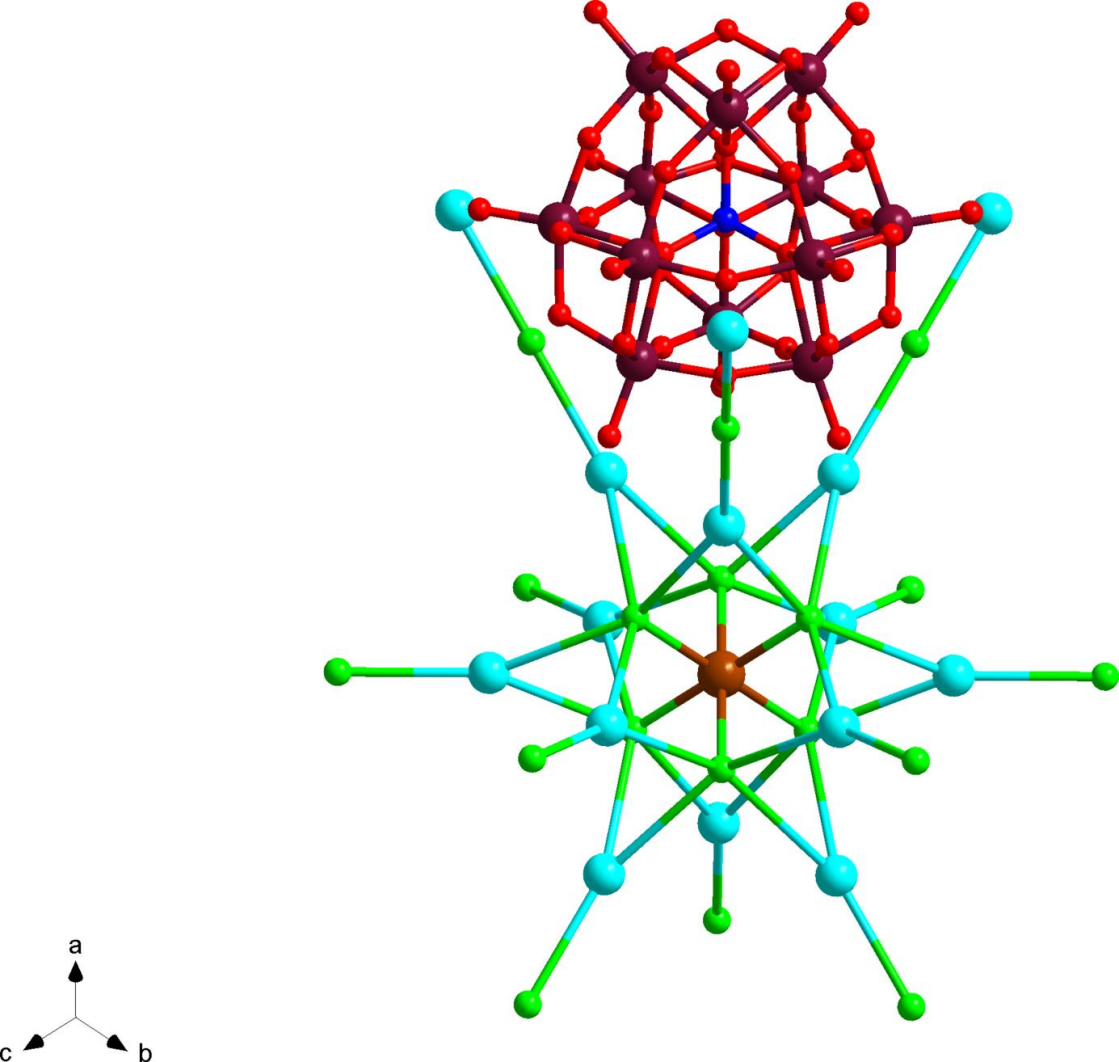
Ball-and-stick representation of [α-PW_12_O_40_]^3−^ and [TlCl_6_]^3−^, showcasing the connectivity with the excess CsCl. W in maroon, O in red, P in blue, Cl in green and Tl in brown.

This new crystal that incorporates thallium(III) into a caesium chloride and phospho­tungstates lattice is fully formulated as Cs_9_(TlCl_6_)(PW_12_O_40_)_2_·9CsCl and crystallizes in the cubic space group *Fm*
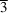
*m* with a volume of 12,166.8 (4) Å^3^, Fig. 1 ▸. The crystal features the parent Keggin structure, [α-PW_12_O_40_]^3−^, which arises from the successive hydrolysis and condensation reactions of [WO_4_]^2−^ in the presence of [PO_4_]^3−^ ions as the pH is lowered (*ca* lower than 7). Briefly, twelve octa­hedral [WO_6_] units can be grouped into four trimer sets [W_3_O_13_]^8−^. Each trimer is linked by the central phosphate anion and then to each other, keeping the overall tetra­hedral symmetry of the central [PO_4_]^3−^. The W—O bond lengths are all consistent with reported values for other POMs (Pope, 1983 ▸): W—OPO_3_ of 2.347 (8) Å, W—O in the range 1.918 (2)–1.942 (4) Å and W=O of 1.713 (10) Å. The asymmetric unit that describes the Keggin ion is thus represented by the tungsten (W1), oxygen (O1, O2, O3, and O4), and phospho­rous (P1) atoms. The tetra­hedral symmetry of the Keggin ion thus arises from the tetra­hedral symmetry of the central phosphate ion (atoms P1/O1–O4). Atom P1 is on the Wyckoff site 8*c*, corresponding to 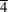
*m*3 symmetry, which then extends to O4 with a Wyckoff site of 32*f* with a symmetry of ·3*m*. The remaining atoms W1, O1, O2, and O3 thus arrange themselves with the same tetra­hedral symmetry, however, now with a Wyckoff site symmetry of ··*m*. The structure further features the sixfold coordinate Tl^3+^ ion, making a [TlCl_6_]^3−^ complex. The arrangement of the complex within the structure is discussed in the next section. Nevertheless, the asymmetric unit that describes the thallium complex is comprised of Tl1 and Cl1 and Wyckoff site symmetries of 4*a* and 24*e*, respectively. The symmetry around Tl1 *i.e.* Wyckoff site 4*a* is *m*
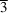
*m*, with Cl1 having 4*mm* symmetry. This results in an octa­hedral complex with six chlorides bound to Tl^3+^. However, the [TlCl_6_]^3−^ ion features slightly longer bond lengths between Tl and Cl of 2.613 (12) Å compared to 2.423 Å in KTlCl_4_ (ICSD 1527421; Glaser, 1980 ▸). What is unusual about the structure is the large excess of CsCl crystallizing – nine CsCl per [TlCl_6_]^3−^ complex. The asymmetric unit only consists of two unique Cs atoms, Cs1 and Cs2 with Wyckoff sites of 48*h* and 24*e*, respectively. These caesium atoms can then thus be thought to coordinate to the other chlorides as well, Cl2, and Cl3. Nevertheless, the excess CsCl becomes significantly important when considering the relative arrangement of the two [PW_12_O_40_]^3−^ and the [TlCl_6_]^3−^ complex, Fig. 2 ▸. All caesium counter-ions are nine-coordinated with Cs—O distances ranging 3.179 (10)–3.221 (7) Å and Cs—Cl ranging from 3.2081 (18)–4.139 (12) Å.

## 3. Supra­molecular features

**Figure 3:**
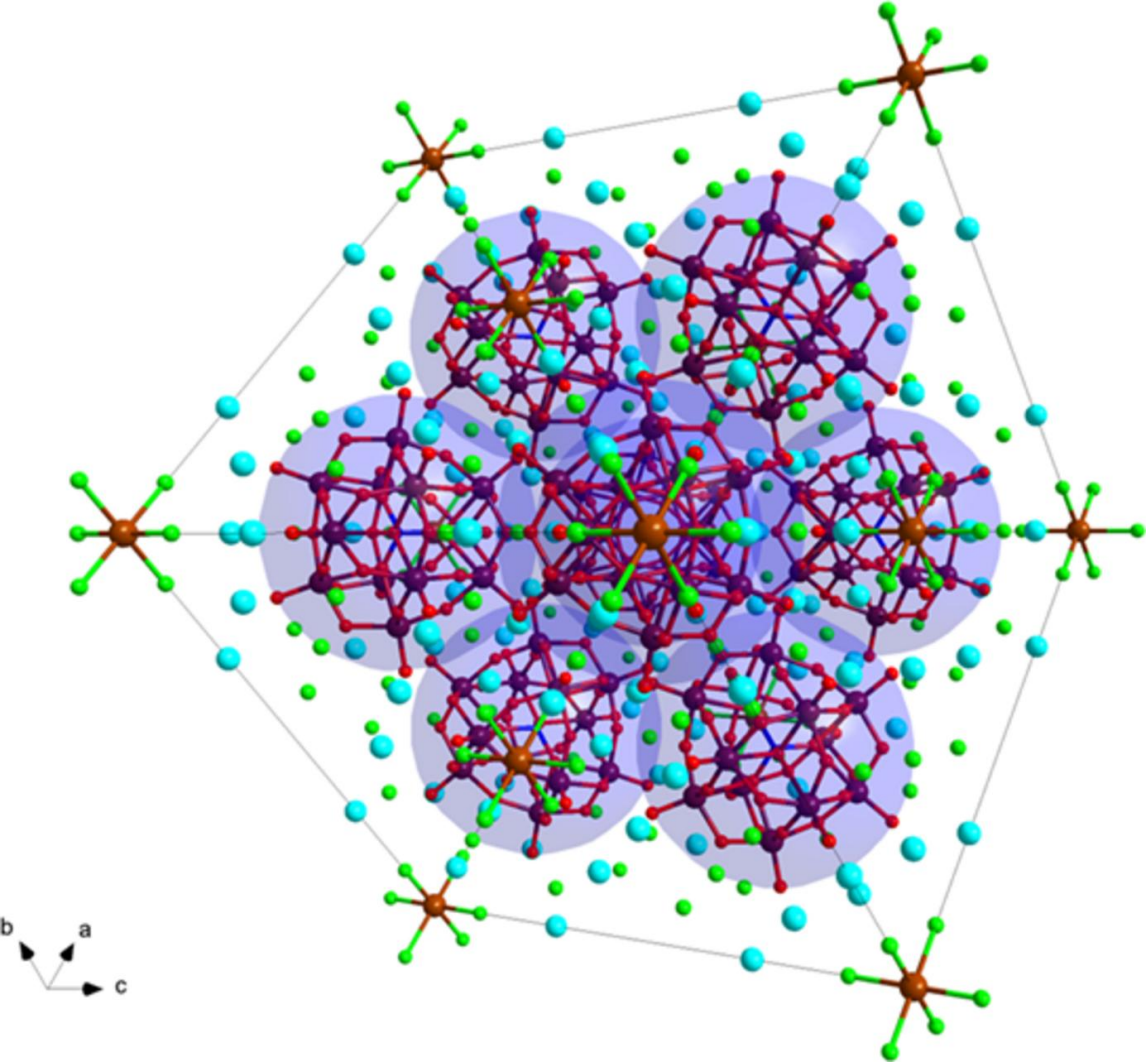
Ball-and-stick representation of the unit cell viewed along (111) showing the cubic close packing of [α-PW_12_O_40_]^3−^ by additionally overlapping the blue spheres to see the ABC layers. The [TlCl_6_]^3−^ ion thus fills half the octa­hedral voids. W in maroon, O in red, P in blue, Cl in green and Tl in brown.

The supra­molecular assembly of the crystal is particularly inter­esting and departs from the typical structures observed with the Keggin ion. The [PW_12_O_40_]^3−^ anion herein behaves like a super-atom. Super-atoms are nano-sized structures that mimic atomic behavior, in particular in the lattice formations (Colliard *et al.*, 2020 ▸). In this structure, the [PW_12_O_40_]^3−^ anion can be thought to arrange itself in a cubic close packing within the unit cell. The Wyckoff letter of *P*1 (8*c*) and the single phospho­rous per Keggin reveals there are eight Keggin ions per unit cell, which is consistent with the face-centered cubic space group *Fm*
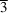
*m.* Caesium counter-ions link all the [PW_12_O_40_]^3−^ anions together, forming an extended framework. As a result of this close cubic packing, the octa­hedral [TlCl_6_]^3−^ ions can fill in the octa­hedral voids left by the cubic close packing of the Keggin ions. Since the synthesis conditions were limited to a 1:2 ratio of Tl^3+^ to [PW_12_O_40_]^3−^, the [TlCl_6_]^3−^ only fills half of the octa­hedral voids, Fig. 3 ▸.

## 4. Database survey

A search of the Cambridge Crystallographic Database (CSD, accessed in April 2024; Groom *et al.*, 2016 ▸) was performed for closely related thallium caesium phospho­tungstates. First, in a unit-cell search [*a* = *b* = *c* = 22.9999 (4) Å, and α = β = γ = 90°, with tolerance of 2% each], with face centering, 29 results were found, none of which contained any tungstates or thallium. With a primitive centering, 95 results were found, again none of which contained any tungstates or thallium. Therefore, a second search was conducted based on the general formula TlPW_12_O_40_ with the option to allow other elements in the mol­ecule and no results were obtained. As such, the search was expanded to another formula search for any structures with W, O, and Tl, none of which consisted of phospho­tungstates and/or thallium compounds. Only one compound containing K, W, O, and Tl was found, but this additionally contains uranium and is not comprised of the Keggin structure (Balboni & Burns, 2014 ▸).

## 5. Synthesis and crystallization

**Figure 4:**
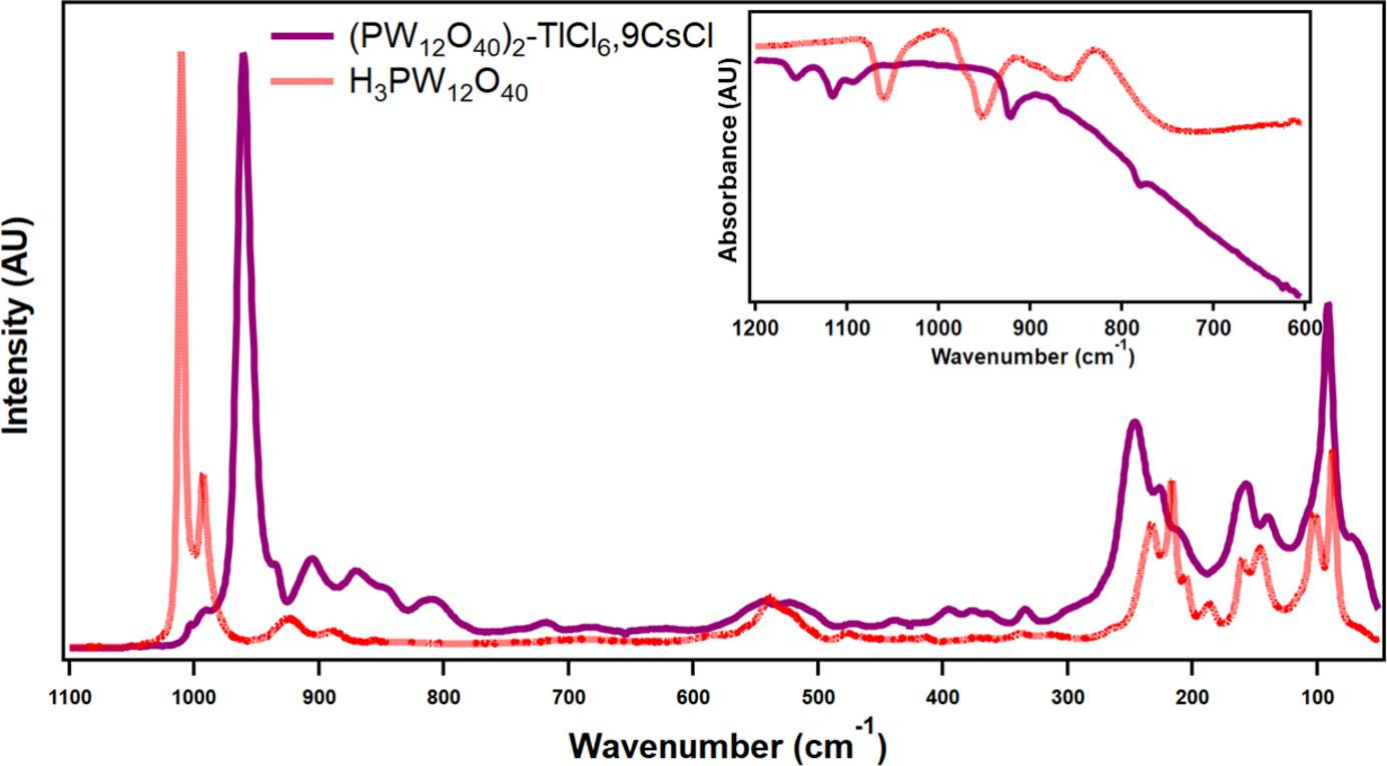
Solid-state Raman and IR (inset) spectra for Cs_9_(TlCl_6_)(PW_12_O_40_)_2_·9CsCl with H_3_PW_12_O_40_ as a comparison.

All materials herein were purchased and used as is, with no need for further purification: NaCl (≥99.9%), NaCH_3_COO (≥99.9%), caesium chloride (>99.99%), Na_2_WO_4_·2H_2_O (≥99%), phospho­ric acid, and thallium trichloride (>99.9%) were purchased from chemical providers (VWR and Millipore Sigma) and used as received. All solutions were prepared using deionized water purified by reverse osmosis cartridge system (**>=** 18.2 MΩ.cm). All experiments were performed in a temperature-controlled room (22°C). Na_9_PW_9_O_34_·7H_2_O was prepared by dissolving 12 g of Na_2_WO_4_·2H_2_O in 15 mL of H_2_O. 0.4 mL of 85% H_3_PO_4_ was added dropwise. Afterwards the pH was adjusted to 7–7.5 with glacial acetic acid (2.25 mL). During the addition, a white solid formed immediately. The solid-solution slurry was left to stir for an hour, after which the solid was filtered under vacuum. [PW_9_O_34_]^9−^ converts to [PW_11_O_39_]^7−^ instantaneously at pH 5.5 (Contant *et al.*, 1990 ▸). A thallium(III) nitrate solution was prepared by dissolving the corresponding Tl(NO_3_)_3_ in 0.1 *M* HCl. After this, the Tl^3+^ solution was added to a 1 mL 200 µ*M* Na_9_PW_9_O_34_·7H_2_O solution in 0.1 *M* acetate buffer at pH 5.5 at a 1:2 stoichi­ometry. For crystallization, 6 *M* CsCl was titrated in 5–50 µL to 1:2 stochiometric solutions (10 to 100 µL, at pH 5.5, 100 m*M* acetate buffer) with a final pH of 5.5 during crystallization. After 1–5 days at ambient conditions, several cube-shaped single crystals of PW_12_-TlCsCl were visible to the naked eye. XRD-quality crystals were then mounted and characterized, while the rest were characterized through Raman microscopy. Raman spectra were collected using a Senterra II confocal Raman microscope (Bruker), equipped with high resolution gratings (1200 lines mm^−1^) and a 532 nm laser source (operated at 15 mW), and a TE-cooled CCD detector. Reported spectra are the average of at least 2–5 different spots per sample, each spot analysis consisting of 2 binned 16 scans. The integration time was set to 2000 ms per scan. No damage to the sample was observed due to the laser irradiation. Infrared spectra were collected using a Cary 630 FTIR instrument (Agilent Technologies) equipped with an attenuated total reflectance (diamond ATR) cell. Selected Raman data (cm^−1^): ν(W=O^*t*^) 961, and ν(O—W—O) 246, 156, and 91; selected IR data (cm^−1^): 1157, 1118, 922, and 782 (Fig. 4 ▸).

## 6. Refinement

Crystal data, data collection and structure refinement details are summarized in Table 1 ▸. All atoms were refined anisotropically. The only issue resulting from the high *Z* for tungsten and Cs was that high residual *Q*-peaks of 10% of *Z* A^−3^ remained (Massa & Gould, 2004 ▸); the highest residual *Q*-peak at 3.9 located at (½, ½, ½) could not reasonably be assigned to any of the elements already present (or those present during synthesis).

## Supplementary Material

Crystal structure: contains datablock(s) I. DOI: 10.1107/S2056989024005565/ox2004sup1.cif

Structure factors: contains datablock(s) I. DOI: 10.1107/S2056989024005565/ox2004Isup2.hkl

CCDC reference: 2361833

Additional supporting information:  crystallographic information; 3D view; checkCIF report

## Figures and Tables

**Table 1:** Experimental details

Crystal data	
Chemical formula	Cs_9_(TlCl_6_)(PW_12_O_40_)_2_·9CsCl
*M*_r_	17765.68
Crystal system, space group	Cubic, *Fm* 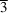 *m*
Temperature (K)	298
*a* (Å)	22.9963 (3)
*V* (Å^3^)	12161.1 (4)
*Z*	2
Radiation type	Mo *K*α
μ (mm^−1^)	29.66
Crystal size (mm)	0.10 × 0.09 × 0.07

Data collection	
Diffractometer	Rigaku Oxford Diffraction, Synergy Custom DW system, Pilatus 300K
Absorption correction	Multi-scan (*CrysAlis PRO*; Rigaku OD, 2019 ▸)
*T*_min_, *T*_max_	0.702, 1.000
No. of measured, independent and observed [*I* > 2σ(*I*)] reflections	6187, 977, 923
*R*_int_	0.026
(sin θ/λ)_max_ (Å^−1^)	0.747

Refinement	
*R*[*F*^2^ > 2σ(*F*^2^)], *wR*(*F*^2^), *S*	0.042, 0.116, 1.20
No. of reflections	977
No. of parameters	47
Δρ_max_, Δρ_min_ (e Å^−3^)	3.92, −4.62

Computer programs: *CrysAlis PRO* (Rigaku OD, 2019 ▸), *SHELXT* (Sheldrick, 2015*a* ▸), *SHELXL2019/3* (Sheldrick, 2015*b* ▸) and *OLEX2* (Dolomanov *et al.*, 2009 ▸).

## supplementary crystallographic information

### Caesium thallium chloride phosphotungstate Crystal data

**Table:**

Cs_9_(TlCl_6_)(PW_12_O_40_)_2_·9CsCl	Mo *K*α radiation, λ = 0.71073 Å
*M**_r_* = 17765.68	Cell parameters from 3426 reflections
Cubic, *F**m*3*m*	θ = 3.8–31.4°
*a* = 22.9963 (3) Å	µ = 29.66 mm^−^^1^
*V* = 12161.1 (4) Å^3^	*T* = 298 K
*Z* = 2	Cube, clear colourless
*F*(000) = 15088	0.10 × 0.09 × 0.07 mm
*D*_x_ = 4.852 Mg m^−^^3^	

### Caesium thallium chloride phosphotungstate Data collection

**Table:**

Rigaku Oxford Diffraction, Synergy Custom DW system, Pilatus 300K diffractometer	923 reflections with *I* > 2σ(*I*)
Detector resolution: 5.8140 pixels mm^-1^	*R*_int_ = 0.026
ω scans	θ_max_ = 32.1°, θ_min_ = 3.9°
Absorption correction: multi-scan (CrysAlisPro; Rigaku OD, 2019)	*h* = −19→33
*T*_min_ = 0.702, *T*_max_ = 1.000	*k* = −18→31
6187 measured reflections	*l* = −30→33
977 independent reflections	

### Caesium thallium chloride phosphotungstate Refinement

**Table:**

Refinement on *F*^2^	47 parameters
Least-squares matrix: full	0 restraints
*R*[*F*^2^ > 2σ(*F*^2^)] = 0.042	*w* = 1/[σ^2^(*F*_o_^2^) + (0.0429*P*)^2^ + 1815.4844*P*] where *P* = (*F*_o_^2^ + 2*F*_c_^2^)/3
*wR*(*F*^2^) = 0.116	(Δ/σ)_max_ = 0.001
*S* = 1.20	Δρ_max_ = 3.92 e Å^−^^3^
977 reflections	Δρ_min_ = −4.62 e Å^−^^3^

### Caesium thallium chloride phosphotungstate Special details

**Table:**

Geometry. All esds (except the esd in the dihedral angle between two l.s. planes) are estimated using the full covariance matrix. The cell esds are taken into account individually in the estimation of esds in distances, angles and torsion angles; correlations between esds in cell parameters are only used when they are defined by crystal symmetry. An approximate (isotropic) treatment of cell esds is used for estimating esds involving l.s. planes.

### Caesium thallium chloride phosphotungstate Fractional atomic coordinates and isotropic or equivalent isotropic displacement parameters (Å^2^)

**Table:**

	*x*	*y*	*z*	*U*_iso_*/*U*_eq_	Occ. (<1)
W1	0.35861 (2)	0.64139 (2)	0.74467 (2)	0.01874 (18)	
Tl1	0.500000	0.500000	1.000000	0.0431 (8)	
Cs1	0.34865 (6)	0.500000	0.84865 (6)	0.0432 (4)	
Cs2	0.500000	0.500000	0.70636 (17)	0.0628 (8)	
P1	0.250000	0.750000	0.750000	0.0151 (19)	
Cl2	0.250000	0.500000	0.750000	0.049 (2)	
Cl1	0.500000	0.500000	0.8864 (5)	0.057 (3)	
O2	0.3797 (4)	0.7015 (3)	0.7985 (3)	0.0178 (16)	
O3	0.3161 (3)	0.6010 (4)	0.6839 (3)	0.0213 (18)	
O1	0.4101 (3)	0.5899 (3)	0.7606 (4)	0.028 (2)	
O4	0.2910 (3)	0.7090 (3)	0.7090 (3)	0.013 (3)	
Cl3	0.3672 (9)	0.500000	0.6328 (9)	0.061 (9)	0.25

### Caesium thallium chloride phosphotungstate Atomic displacement parameters (Å^2^)

**Table:**

	*U*^11^	*U*^22^	*U*^33^	*U*^12^	*U*^13^	*U*^23^
W1	0.0172 (2)	0.0172 (2)	0.0219 (3)	0.00357 (17)	−0.00094 (12)	0.00094 (12)
Tl1	0.0431 (8)	0.0431 (8)	0.0431 (8)	0.000	0.000	0.000
Cs1	0.0501 (6)	0.0295 (7)	0.0501 (6)	0.000	−0.0033 (7)	0.000
Cs2	0.0426 (8)	0.0426 (8)	0.103 (3)	0.000	0.000	0.000
P1	0.0151 (19)	0.0151 (19)	0.0151 (19)	0.000	0.000	0.000
Cl2	0.065 (4)	0.016 (3)	0.065 (4)	0.000	0.013 (5)	0.000
Cl1	0.060 (4)	0.060 (4)	0.051 (6)	0.000	0.000	0.000
O2	0.016 (4)	0.019 (2)	0.019 (2)	−0.003 (2)	0.003 (2)	0.002 (3)
O3	0.024 (3)	0.016 (4)	0.024 (3)	0.001 (2)	0.004 (4)	−0.001 (2)
O1	0.027 (3)	0.027 (3)	0.029 (5)	0.010 (4)	0.001 (3)	−0.001 (3)
O4	0.013 (3)	0.013 (3)	0.013 (3)	0.000 (3)	0.000 (3)	0.000 (3)
Cl3	0.078 (14)	0.025 (11)	0.078 (14)	0.000	0.034 (17)	0.000

### Caesium thallium chloride phosphotungstate Geometric parameters (Å, º)

**Table:**

W1—Cs1	4.0425 (8)	Cs1—O2^ii^	3.211 (9)
W1—Cs1^i^	4.0425 (8)	Cs1—O1	3.221 (7)
W1—O2^ii^	1.918 (2)	Cs1—O1^ii^	3.221 (7)
W1—O2	1.918 (2)	Cs1—O1^x^	3.221 (7)
W1—O3	1.942 (4)	Cs1—O1^ix^	3.221 (7)
W1—O3^iii^	1.942 (4)	Cs2—Cl1	4.139 (12)
W1—O1	1.713 (10)	Cs2—O1^x^	3.179 (10)
W1—O4	2.347 (8)	Cs2—O1	3.179 (10)
Tl1—Cl1^iv^	2.613 (12)	Cs2—O1^xi^	3.179 (10)
Tl1—Cl1^v^	2.613 (12)	Cs2—O1^xii^	3.179 (10)
Tl1—Cl1^vi^	2.613 (12)	Cs2—Cl3^xiii^	3.492 (9)
Tl1—Cl1	2.613 (12)	Cs2—Cl3	3.492 (9)
Tl1—Cl1^vii^	2.613 (12)	Cs2—Cl3^xii^	3.492 (9)
Tl1—Cl1^viii^	2.613 (12)	Cs2—Cl3^iii^	3.492 (9)
Cs1—Cl2	3.2081 (18)	P1—O4	1.633 (14)
Cs1—Cl1^v^	3.587 (3)	P1—O4^xiv^	1.633 (14)
Cs1—Cl1	3.587 (3)	P1—O4^xv^	1.633 (14)
Cs1—O2^ix^	3.211 (9)	P1—O4^xvi^	1.633 (14)
			
Cs1^i^—W1—Cs1	75.01 (5)	O1^x^—Cs1—O1^ii^	164.5 (3)
O2^ii^—W1—Cs1	51.2 (3)	O1^ix^—Cs1—O1^ii^	79.9 (3)
O2^ii^—W1—Cs1^i^	102.2 (3)	Cs1^xii^—Cs2—Cs1	93.54 (8)
O2—W1—Cs1	102.2 (3)	Cs1^i^—Cs2—Cs1^xii^	62.02 (5)
O2—W1—Cs1^i^	51.2 (3)	Cs1^i^—Cs2—Cs1	62.02 (5)
O2—W1—O2^ii^	87.0 (5)	Cl1—Cs2—Cs1^xii^	46.77 (4)
O2—W1—O3	159.5 (3)	Cl1—Cs2—Cs1^i^	46.77 (4)
O2—W1—O3^iii^	88.9 (4)	Cl1—Cs2—Cs1	46.77 (4)
O2^ii^—W1—O3	88.9 (4)	O1—Cs2—Cs1^xii^	101.85 (18)
O2^ii^—W1—O3^iii^	159.5 (3)	O1—Cs2—Cs1^i^	42.05 (13)
O2—W1—O4	85.2 (3)	O1^xi^—Cs2—Cs1	101.84 (18)
O2^ii^—W1—O4	85.2 (3)	O1^xii^—Cs2—Cs1	101.84 (18)
O3^iii^—W1—Cs1^i^	90.7 (3)	O1—Cs2—Cs1	42.05 (13)
O3^iii^—W1—Cs1	149.1 (2)	O1^xi^—Cs2—Cs1^i^	42.05 (13)
O3—W1—Cs1	90.7 (3)	O1^x^—Cs2—Cs1	42.05 (12)
O3—W1—Cs1^i^	149.1 (2)	O1^x^—Cs2—Cs1^i^	101.85 (18)
O3^iii^—W1—O3	87.9 (5)	O1^xii^—Cs2—Cs1^i^	101.85 (18)
O3—W1—O4	74.4 (3)	O1^xi^—Cs2—Cs1^xii^	42.05 (12)
O3^iii^—W1—O4	74.4 (3)	O1^xii^—Cs2—Cs1^xii^	42.05 (13)
O1—W1—Cs1	50.0 (2)	O1^x^—Cs2—Cs1^xii^	101.85 (18)
O1—W1—Cs1^i^	50.0 (2)	O1^x^—Cs2—Cl1	66.9 (2)
O1—W1—O2	100.7 (4)	O1^xii^—Cs2—Cl1	66.9 (2)
O1—W1—O2^ii^	100.7 (4)	O1—Cs2—Cl1	66.9 (2)
O1—W1—O3	99.9 (3)	O1^xi^—Cs2—Cl1	66.9 (2)
O1—W1—O3^iii^	99.9 (3)	O1—Cs2—O1^xi^	81.15 (15)
O1—W1—O4	171.9 (4)	O1^xi^—Cs2—O1^xii^	81.15 (15)
O4—W1—Cs1	134.59 (15)	O1—Cs2—O1^xii^	133.8 (4)
O4—W1—Cs1^i^	134.59 (15)	O1—Cs2—O1^x^	81.15 (15)
Cl1^vi^—Tl1—Cl1^vii^	180.0	O1^xi^—Cs2—O1^x^	133.8 (4)
Cl1^v^—Tl1—Cl1^iv^	180.0	O1^x^—Cs2—O1^xii^	81.15 (15)
Cl1^v^—Tl1—Cl1	90.000 (1)	O1^xi^—Cs2—Cl3^xii^	67.7 (4)
Cl1^vi^—Tl1—Cl1^viii^	90.000 (1)	O1^xi^—Cs2—Cl3^xiii^	139.38 (7)
Cl1^vii^—Tl1—Cl1	90.000 (1)	O1^xii^—Cs2—Cl3	139.38 (7)
Cl1^vi^—Tl1—Cl1^v^	90.000 (1)	O1^x^—Cs2—Cl3^xii^	139.38 (6)
Cl1^viii^—Tl1—Cl1^iv^	90.000 (2)	O1^xii^—Cs2—Cl3^xiii^	67.7 (4)
Cl1^vii^—Tl1—Cl1^v^	90.000 (3)	O1—Cs2—Cl3^iii^	67.7 (4)
Cl1^vi^—Tl1—Cl1	90.000 (1)	O1—Cs2—Cl3^xiii^	139.38 (7)
Cl1^viii^—Tl1—Cl1^v^	90.000 (3)	O1^xi^—Cs2—Cl3^iii^	67.7 (4)
Cl1^viii^—Tl1—Cl1	180.0	O1^x^—Cs2—Cl3^xiii^	67.7 (4)
Cl1^vi^—Tl1—Cl1^iv^	90.000 (3)	O1—Cs2—Cl3^xii^	139.38 (7)
Cl1^iv^—Tl1—Cl1	90.000 (1)	O1^xii^—Cs2—Cl3^xii^	67.7 (4)
Cl1^vii^—Tl1—Cl1^iv^	90.000 (1)	O1^x^—Cs2—Cl3	67.7 (4)
Cl1^vii^—Tl1—Cl1^viii^	90.000 (1)	O1^x^—Cs2—Cl3^iii^	139.38 (7)
W1^ix^—Cs1—W1^x^	54.562 (17)	O1—Cs2—Cl3	67.7 (4)
W1—Cs1—W1^ix^	135.56 (5)	O1^xi^—Cs2—Cl3	139.38 (7)
W1—Cs1—W1^x^	107.09 (3)	O1^xii^—Cs2—Cl3^iii^	139.38 (7)
Cl2—Cs1—W1^ix^	67.78 (2)	Cl3—Cs2—Cs1^xii^	165.7 (5)
Cl2—Cs1—W1^x^	67.78 (2)	Cl3^xii^—Cs2—Cs1	165.7 (5)
Cl2—Cs1—W1	67.78 (2)	Cl3—Cs2—Cs1	72.2 (5)
Cl2—Cs1—Cl1	148.99 (18)	Cl3^xiii^—Cs2—Cs1^xii^	109.4 (3)
Cl2—Cs1—Cl1^v^	148.99 (18)	Cl3^xii^—Cs2—Cs1^i^	109.4 (3)
Cl2—Cs1—O2^ix^	59.49 (15)	Cl3^iii^—Cs2—Cs1^xii^	109.4 (3)
Cl2—Cs1—O2^ii^	59.49 (15)	Cl3^iii^—Cs2—Cs1	109.4 (3)
Cl2—Cs1—O1	82.27 (15)	Cl3^xii^—Cs2—Cs1^xii^	72.2 (5)
Cl2—Cs1—O1^x^	82.27 (15)	Cl3^iii^—Cs2—Cs1^i^	72.2 (5)
Cl2—Cs1—O1^ii^	82.27 (15)	Cl3—Cs2—Cs1^i^	109.4 (3)
Cl2—Cs1—O1^ix^	82.27 (15)	Cl3^xiii^—Cs2—Cs1^i^	165.7 (5)
Cl1^v^—Cs1—W1	124.07 (5)	Cl3^xiii^—Cs2—Cs1	109.4 (3)
Cl1—Cs1—W1^x^	95.05 (11)	Cl3—Cs2—Cl1	119.0 (5)
Cl1^v^—Cs1—W1^ix^	95.05 (11)	Cl3^iii^—Cs2—Cl1	119.0 (5)
Cl1—Cs1—W1^ix^	124.07 (5)	Cl3^xiii^—Cs2—Cl1	119.0 (5)
Cl1—Cs1—W1	95.05 (11)	Cl3^xii^—Cs2—Cl1	119.0 (5)
Cl1^v^—Cs1—W1^x^	124.07 (5)	Cl3^iii^—Cs2—Cl3^xii^	76.4 (4)
Cl1—Cs1—Cl1^v^	62.0 (4)	Cl3—Cs2—Cl3^iii^	76.4 (4)
O2^ix^—Cs1—W1^x^	27.76 (3)	Cl3—Cs2—Cl3^xiii^	76.4 (4)
O2^ii^—Cs1—W1^ix^	120.06 (13)	Cl3^xiii^—Cs2—Cl3^xii^	76.4 (4)
O2^ix^—Cs1—W1	120.06 (13)	Cl3—Cs2—Cl3^xii^	122.1 (10)
O2^ii^—Cs1—W1^x^	120.06 (13)	Cl3^xiii^—Cs2—Cl3^iii^	122.1 (10)
O2^ii^—Cs1—W1	27.76 (3)	O4^xvi^—P1—O4^xiv^	109.471 (3)
O2^ix^—Cs1—W1^ix^	27.76 (3)	O4—P1—O4^xiv^	109.471 (1)
O2^ix^—Cs1—Cl1	115.80 (13)	O4^xiv^—P1—O4^xv^	109.471 (2)
O2^ix^—Cs1—Cl1^v^	115.80 (13)	O4—P1—O4^xv^	109.5
O2^ii^—Cs1—Cl1^v^	115.80 (13)	O4^xvi^—P1—O4^xv^	109.5
O2^ii^—Cs1—Cl1	115.80 (13)	O4—P1—O4^xvi^	109.471 (6)
O2^ix^—Cs1—O2^ii^	119.0 (3)	Cs1^xvii^—Cl2—Cs1	180.0
O2^ix^—Cs1—O1^ix^	51.58 (18)	Tl1—Cl1—Cs1^i^	103.99 (18)
O2^ix^—Cs1—O1	119.00 (17)	Tl1—Cl1—Cs1	103.99 (18)
O2^ii^—Cs1—O1^x^	119.00 (17)	Tl1—Cl1—Cs1^xii^	103.99 (18)
O2^ix^—Cs1—O1^ii^	119.00 (17)	Tl1—Cl1—Cs1^vii^	103.99 (18)
O2^ii^—Cs1—O1^ii^	51.58 (18)	Tl1—Cl1—Cs2	180.0
O2^ii^—Cs1—O1	51.58 (18)	Cs1^vii^—Cl1—Cs1^i^	152.0 (4)
O2^ix^—Cs1—O1^x^	51.58 (18)	Cs1^i^—Cl1—Cs1^xii^	86.65 (8)
O2^ii^—Cs1—O1^ix^	119.00 (17)	Cs1—Cl1—Cs1^i^	86.65 (8)
O1—Cs1—W1^x^	96.87 (16)	Cs1—Cl1—Cs1^vii^	86.65 (8)
O1^ix^—Cs1—W1	144.24 (15)	Cs1—Cl1—Cs1^xii^	152.0 (4)
O1^x^—Cs1—W1^x^	24.05 (18)	Cs1^vii^—Cl1—Cs1^xii^	86.65 (8)
O1^ix^—Cs1—W1^ix^	24.05 (18)	Cs1—Cl1—Cs2	76.01 (18)
O1^ix^—Cs1—W1^x^	77.22 (18)	Cs1^xii^—Cl1—Cs2	76.01 (18)
O1^ii^—Cs1—W1^x^	144.24 (15)	Cs1^vii^—Cl1—Cs2	76.01 (18)
O1^ii^—Cs1—W1^ix^	96.87 (16)	Cs1^i^—Cl1—Cs2	76.01 (18)
O1—Cs1—W1	24.05 (18)	W1—O2—W1^xviii^	150.0 (5)
O1^x^—Cs1—W1	96.87 (16)	W1—O2—Cs1^i^	101.0 (3)
O1—Cs1—W1^ix^	144.24 (15)	W1^xviii^—O2—Cs1^i^	101.0 (3)
O1^x^—Cs1—W1^ix^	77.22 (18)	W1^xix^—O3—W1	119.7 (4)
O1^ii^—Cs1—W1	77.22 (18)	W1—O1—Cs1^i^	105.9 (3)
O1^x^—Cs1—Cl1^v^	120.27 (19)	W1—O1—Cs1	105.9 (3)
O1—Cs1—Cl1	74.1 (2)	W1—O1—Cs2	144.6 (5)
O1^x^—Cs1—Cl1	74.1 (2)	Cs1^i^—O1—Cs1	99.7 (3)
O1^ii^—Cs1—Cl1^v^	74.1 (2)	Cs2—O1—Cs1	96.6 (2)
O1^ix^—Cs1—Cl1	120.27 (19)	Cs2—O1—Cs1^i^	96.6 (2)
O1^ix^—Cs1—Cl1^v^	74.1 (2)	W1—O4—W1^iii^	91.4 (4)
O1^ii^—Cs1—Cl1	120.27 (19)	W1^xix^—O4—W1^iii^	91.4 (4)
O1—Cs1—Cl1^v^	120.27 (19)	W1—O4—W1^xix^	91.4 (4)
O1^ii^—Cs1—O1	98.0 (4)	P1—O4—W1^iii^	124.3 (3)
O1^ix^—Cs1—O1^x^	98.0 (3)	P1—O4—W1^xix^	124.3 (3)
O1^x^—Cs1—O1	79.9 (3)	P1—O4—W1	124.3 (3)
O1^ix^—Cs1—O1	164.5 (3)	Cs2^xx^—Cl3—Cs2	147.9 (10)
			
Cs1—W1—O1—Cs1^i^	−105.3 (5)	O3—W1—O1—Cs1	−82.6 (4)
Cs1^i^—W1—O1—Cs1	105.3 (5)	O3^iii^—W1—O1—Cs2	−44.8 (3)
Cs1^i^—W1—O1—Cs2	−127.4 (2)	O3—W1—O1—Cs2	44.8 (3)
Cs1—W1—O1—Cs2	127.4 (2)	O4^xvi^—P1—O4—W1^xix^	180.000 (1)
O2—W1—O1—Cs1^i^	−8.2 (4)	O4^xv^—P1—O4—W1	−60.000 (1)
O2—W1—O1—Cs1	97.1 (3)	O4^xv^—P1—O4—W1^xix^	60.000 (1)
O2^ii^—W1—O1—Cs1^i^	−97.1 (3)	O4^xiv^—P1—O4—W1^xix^	−60.000 (1)
O2^ii^—W1—O1—Cs1	8.2 (4)	O4^xvi^—P1—O4—W1^iii^	−60.000 (1)
O2^ii^—W1—O1—Cs2	135.5 (2)	O4^xiv^—P1—O4—W1	180.000 (1)
O2—W1—O1—Cs2	−135.5 (2)	O4^xiv^—P1—O4—W1^iii^	60.000 (1)
O3^iii^—W1—O1—Cs1	−172.1 (3)	O4^xv^—P1—O4—W1^iii^	180.000 (1)
O3—W1—O1—Cs1^i^	172.1 (3)	O4^xvi^—P1—O4—W1	60.000 (2)
O3^iii^—W1—O1—Cs1^i^	82.6 (4)		

Symmetry codes: (i) *y*, −*z*+3/2, *x*+1/2; (ii) *z*−1/2, −*x*+1, −*y*+3/2; (iii) −*y*+1, *z*, −*x*+1; (iv) −*z*+3/2, −*x*+1, −*y*+3/2; (v) *z*−1/2, *x*, *y*+1/2; (vi) −*y*+1, −*z*+3/2, −*x*+3/2; (vii) *y*, *z*−1/2, *x*+1/2; (viii) −*x*+1, −*y*+1, −*z*+2; (ix) *z*−1/2, *x*, −*y*+3/2; (x) *x*, −*y*+1, *z*; (xi) −*x*+1, *y*, *z*; (xii) −*x*+1, −*y*+1, *z*; (xiii) −*y*+1, −*z*+1, −*x*+1; (xiv) −*x*+1/2, −*y*+3/2, *z*; (xv) −*x*+1/2, *y*, −*z*+3/2; (xvi) *x*, −*y*+3/2, −*z*+3/2; (xvii) −*x*+1/2, −*y*+1, −*z*+3/2; (xviii) −*y*+1, −*z*+3/2, *x*+1/2; (xix) −*z*+1, −*x*+1, *y*; (xx) −*z*+1, −*x*+1, −*y*+1.
